# Comparison of Clay Ceramsite and Biodegradable Polymers as Carriers in Pack-bed Biofilm Reactor for Nitrate Removal

**DOI:** 10.3390/ijerph16214184

**Published:** 2019-10-29

**Authors:** Qian Zhang, Xue Chen, Heng Wu, Wandong Luo, Xiangyang Liu, Li Feng, Tiantao Zhao

**Affiliations:** 1 School of Chemistry and Chemical Engineering, Chongqing University of Technology, Chongqing 400054, China; zhangqianswu2005@163.com (Q.Z.); chenxuehur@163.com (X.C.); wuhengdyx@163.com (H.W.); lwd265588@163.com (W.L.); liuxy@2017.cqut.edu.cn (X.L.); 2 Chongqing Academy of Environmental Science, Chongqing 401147, China; fenglihky@163.com

**Keywords:** solid-phase denitrification, conventional denitrification, polycaprolactone (PCL), ceramsite filter, nitrate removal

## Abstract

In recent years, there is a trend of low C/N ratio in municipal domestic wastewater, which results in serious problems for nitrogen removal from wastewater. The addition of an external soluble carbon source has been the usual procedure to achieve denitrification. However, the disadvantage of this treatment process is the need of a closed, rather sophisticated and costly process control as well as the risk of overdosing. Solid-phase denitrification using biodegradable polymers as biofilm carrier and carbon source was considered as an attractive alternative for biological denitrification. The start-up time of the novel process using PCL (polycaprolactone) as biofilm carrier and carbon source was comparable with that of conventional process using ceramsite as biofilm carrier and acetate as carbon source. Further, the solid-phase denitrification process showed higher nitrogen removal efficiency under shorter hydraulic retention time (HRT) and low carbon to nitrogen (C/N) ratio since the biofilm was firmly attached to the clear pores on the surface of PCL carriers and in this process bacteria that could degrade PCL carriers to obtain electron donor for denitrification was found. In addition, solid-phase denitrification process had a stronger resistance of shock loading than that in conventional process. This study revealed, for the first time, that the physical properties of the biodegradable polymer played a vital role in denitrification, and the different microbial compositions of the two processes was the main reason for the different denitrification performances under low C/N ratio.

## 1. Introduction

Secondary effluent, which is produced from wastewater treatment plants (WWTPs) has to be further treated, due to increasing emission standards and water scarcity. Considering the feature of low turbidity and oligotrophic secondary effluent, biofiltration processes such as ceramsite grains, granular activated carbon and quartz sand were widely employed in the advanced treatment [1,2,3]. Due to limited organic carbon source in secondary effluent, denitrification of secondary effluent is usually achieved by extra organic carbon addition (e.g., acetic acid, sodium acetate, glucose, ethanol, etc.) [4]. However, the disadvantages of this treatment process are the necessity of a closed, sophisticated, and costly control process, the risk of overdosing, and the need for in-depth knowledge about the operation of this biological system [5,6]. Biodegradable polymers (BDPs), which act as both an energy source and biofilm carrier, have sparked great interest in the scientific community [7,8,9,10,11,12]. In contrast to conventional treatment units, denitrification with biodegradable polymers facilitates operation. Solid-phase denitrification can effectively avoid process risk resulted from carbon addition (i.e., excess or insufficient) since denitrification of solid organic matter is only realized via enzymatic attack [13]. 

Previous studies mainly focused on materials selection [14,15] and the determination of specific nitrogen removal efficiencies of different BDPs [8,12,16]. However, comprehensive solid-phase denitrification processes are lacking and the results vary with the type [7,17,18,19,20], structure [21,22] and molecular weight [23] of BDPs. Rodrigues et al. [22] found that the same type of BDPs showed different performance on volumetric nitrate removal rate when comparing with the previous literature [5,24] since the BDPs employed in the different works have different physical-chemical properties. Thus, a direct results comparison between solid-phase denitrification and conventional denitrification processes for the different denitrification performances between the two processes during the start-up period and under adverse running conditions (i.e., low carbon to nitrogen (C/N) ratio, short hydraulic retention time (HRT) and continuous shock nitrate loadings) is essential.

In the preliminary experiment, the effects of the type, structure and molecular weight of BDPs on nitrogen removal efficiency was studied. BDPs (PCL) were chosen in this work attributing to the quantities of clear pores on its irregular and rough surface that can provide growth space for the biofilms and protect the attachment of biofilms. This led to the best denitrification performances. The objective of the present study was to investigate the nitrogen removal efficiency of solid-phase denitrification process compared to the conventional denitrification process. The denitrification performances of the two processes during the start-up period were studied and the effect of operation conditions (i.e., HRT, C/N ratio and shock nitrate loading) on denitrification was investigated. The present paper would be useful for researchers and engineers in the field of nitrogen removal from water and wastewater.

## 2. Materials and Methods

### 2.1. Materials

Polycaprolactone (PCL) and clay ceramsite carriers collected from Shenzhen Esun Industrial Co. Ltd. and Jiangxi Pingxiang Sanhe Ceramics Co., Ltd. were used in this study. Flotation is always a useful method to treat solid particles [25,26]. Screening for equal mass carriers was performed with this general method to make sure all the carriers could be firmly fixed at the reactor. The physico-chemical parameters are presented in Table 1.

In the first stage of start-up, domestic wastewater collected from Chongqing University was used as the influent of the reactors. The domestic wastewater contained 148–185 mg/L of COD_Cr_, 26.7–56.8 mg/L of NH_4_^+^-N, and 28.6–70.2 mg/L of TN. In the second stage of start-up and subsequent tests, synthetic wastewater was used to simulate the composition of nitrate-contaminated wastewater. The concentration of COD_Cr_, NO3−-N and PO_4_−-P in synthetic wastewater was controlled by adding sodium acetate, NaNO_3_ and K_2_HPO_4_. During the experiment, the DO and pH value in synthetic water were not monitored.

Activated sludge collected from anoxic phase of the anaerobic/anoxic/oxic (A^2^/O process) of Sewage Plant (Yongchuan, China) was used as seed source for denitrification of continuous experiment. The characteristics of the activated sludge were as follows: 80% of sludge volume (SV) 30, 123.4 mL/g of sludge volume index (SVI), 5.6 g/L of mixed liquor suspended solids (MLSS), and 3.4 g/L of mixed liquor volatile suspended solids (MLVSS).

### 2.2. Experimental Apparatus

**Figure 1:**
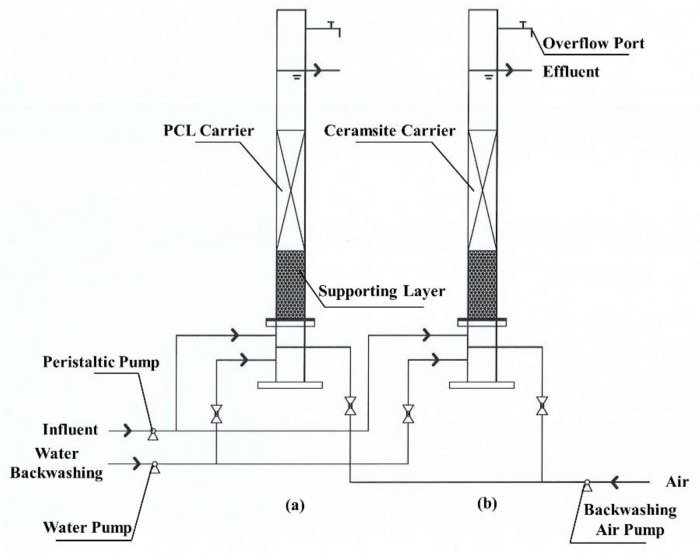
Device scheme: (**a**) Reactor 1; and (**b**) Reactor 2.

The process diagram of experimental setup is shown in Figure 1. Two denitrification biofilters, namely Reactor 1 packed with PCL carrier and Reactor 2 packed with ceramsite carrier, were installed on a laboratory scale. The reactors were two Plexiglas cylinders with 80 mm inner diameter and 1300 mm length. The packing height of PCL and ceramsite in Reactors 1 and 2 was 27 cm (1.4 kg) and 27 cm (2.3 kg), respectively. The effective volume of liquid for each reactor was 3.5 L and the flow rate of influent was adjusted with a peristaltic pump.

### 2.3. Experiment Procedures

Inoculation biofilm formation method was used in the start-up of the reactors. The start-up of the reactors was divided into two stages. In the first stage, 1 L of seeds sludge was added to each reactor filled with domestic sewage wastewater and static incubated for 3 days. In the second stage, synthetic wastewater was continuously fed to the reactors at low flow rate (24.18 L/d). Nitrate of the influent was enhanced stepwise to realize the domestication of denitrifying bacteria. In this stage, two reactors were operated at the HRT of 3.5 h, and the COD_Cr_ concentration of the influent was maintained at constant 80 mg/L. When the start-up of the two reactors was completed, dissolved organic matter in the effluent of the two reactors was characterized by three-dimension excitation-emission matrix (EEM) fluorescence spectroscopy.

The mass balance of total organic carbon (TOC) in solid-phase denitrification biofilter using PCL as biofilm support and carbon source was studied to explore the consumption rate and related organic carbon contribution to denitrification. The influent NO3−N was maintained at 50 mg/L, and no external carbon source was added. The organic carbon release from PCL including physical dissolution (*C_p_*) and biological degradation (*C_b_*), and the consumption of released organic carbon from PCL consisted of denitrification consumption (*C_d_*), dissolved oxygen consumption (*C_o_*) and TOC residual in the effluent (*C_r_*). Since the NO_2_−-N in the effluent was at a very low level and the TOC consumed for cell synthesis was also neglected, the mass balance of PCL in solid-phase denitrification biofilter can be described as Equation (1):

(1) $$C_{b}=C_{d}+C_{o}+C_{r}-C_{P}$$

(2) $$\left(C_{6}H_{10}O_{2}\right)n+6{\mathrm{nNO}}_{3}^{-}\to 3{\mathrm{nN}}_{2}+6{\mathrm{nHCO}}_{3}^{-}+5{\mathrm{nH}}_{2}O$$

(3) $$\left(C_{6}H_{10}O_{2}\right)n+7.5{\mathrm{nO}}_{2}\to 6{\mathrm{nCO}}_{2}+5{\mathrm{nH}}_{2}O$$

where *C_d_* can be calculated through stoichiometric Equation (2); *C_o_* can be calculated by stoichiometric Equation (3); *C_r_* can be calculated according to the influent and effluent TOC concentration; and *C_p_* can be calculated on the basis of filling amount of PCL in the reactor (1.4 kg) and the release rate of PCL in distilled water (0.13 mg·g^−1^·d^−1^). PCL is formed by the polymerization of *ε*-caprolactone (C_6_H_10_O_2_), therefore, PCL in stoichiometric Equation (2) was presented as (C_6_H_10_O_2_)_n_, and the theoretical TOC value of 1 g PCL is 0.63 g.

After a stable denitrification performance was obtained, the influence of HRT, C/N ratio and nitrate shock loading on the denitrification performance of the reactors were investigated. During the HRT test, PCL carriers were taken from Reactor 1 when stable denitrification performance was achieved at a flow rate of 58.3 mL/min (HRT = 1 h), and biofilm attached to the carriers and the raw PCL carriers were examined by an environmental scanning electron microscope (ESEM) (Quanta 200 FEG, Hillsboro, USA). During the C/N ratio test, PCL and ceramsite carriers were taken from the two reactors when stable denitrification rate was obtained at a C/N ratio of 0.9. The biofilm formed on the two carriers expressed as volatile suspended solids (VSS) was quantified, and the samples collected from biofilm attached on the surface of PCL and ceramsite carriers were analyzed by using PCR-denaturing gradient gel electrophoresis (DGGE) technique.

### 2.4. Analytical Methods

Samples collected from the inlet and outlet of the two reactors was filtered through 0.45 μm membrane before analysis. NO3−-N was determined by UV-spectrophotometer (Shimadzu UVmini-1240, Kyoto, Japan) at 220 and 275 nm. Total nitrogen (TN) was measured by oxidizing all the nitrogen components to nitrate nitrogen using alkaline potassium persulfate. NO_2_−-N was detected according to naphthylethylenediamine hydrochloride spectrophotometry method with visible spectrophotometer (Spectrum SP-721E, Shenzhen, China). COD_Cr_ concentration of the influent and effluent were determined by Portable COD Tester (Hach DR1010, Loveland, USA). Fluorescence EEM measurements were conducted by using a fluorescence spectrophotometer (Hitachi F-700, Tokyo, Japan). The biomass contents on the surface of the two carriers were quantified according to Jin et al. [27] The methods used for PCR-DGGE analysis were the same as those described previously by Dong et al. [28].

## 3. Results and Discussions

### 3.1. Denitrification Performance During the Start-up Period

**Figure 2:**
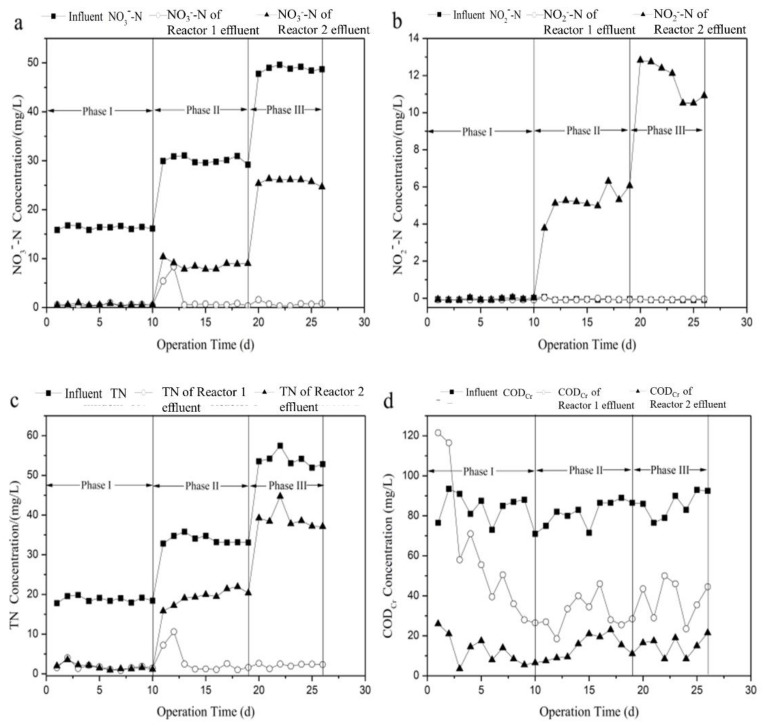
Variation of effluent: (**a**) nitrate; (**b**) nitrite; (**c**) TN; and (**d**) COD_Cr_ concentration of the two processes with time during start-up period.

To investigate the effects of the influent nitrate concentration on the performances of denitrification and bio-film cultivation during the start-up period, variations in the effluent of the two reactors of nitrite, TN and COD_Cr_ concentrations were measured and the results are shown in Figure 2. The start-up of the reactor was divided into three phases according to influent nitrate concentration. COD_Cr_ concentration of influent was maintained at constant 80 mg/L, and HRT was set to 3.5 h. In the initial 10 days, high-efficiency denitrification ability and stable water quality for effluent could be realized when the initial NO3−-N concentration of influent was 16 mg/L. The average concentrations for NO3−-N and TN of the effluent of Reactors 1 and 2 were 0.5 mg/L and 1.8 mg/L, and 0.6 mg/L and 1.8 mg/L (Figure 2a,c), respectively, and no nitrite was detected in the effluent (Figure 2b), which indicated that both reactors had the same denitrification performance attributing to sufficient the organic carbon source in the influent. From Day 12, the nitrate loading increased to 30 mg/L; nitrate concentration in the effluent of Reactor 1 first increased sharply and then decreased to the previous level at the second day. Subsequently, NO3−-N and TN concentration of the effluent of Reactor 1 were almost unchanged, although nitrate loading was increased to 50 mg/L on the 19th day, and no nitrite was detected. In Reactor 2, however, NO3−-N and TN in the effluent increased with increasing nitrate load, and accumulation of nitrite could also be clearly observed. From Day 11, nitrate in the effluent started to decrease (Figure 2c), which indicated that biofilm on ceramsite tended to mature. Boley et al. [5] reported that the point with the steepest negative slope of the nitrate concentration versus time curve is an indication of the unit operating at its maximum. Thus, it can be concluded that the lag-time (adaptive phase about denitrifying microorganisms) of Reactor 1 (12 days) was comparable with that of Reactor 2 (11 days), which was consistent with the previous studies. This demonstrated equivalent performance in the start-up time between modified biodegradable biofilm reactor and the conventional biofilm reactor (ethanol as carbon source) [29]. 

**Figure 3:**
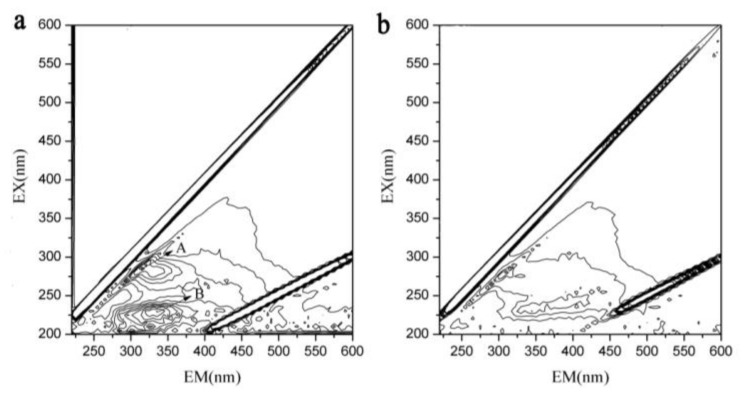
3D-EEM of the effluent from: (**a**) Reactor 1; and (**b**) Reactor 2.

As is shown in Figure 2d, COD_Cr_ concentration in the effluent of Reactors 1 and 2 was high. Therefore, 3D-EEM was used to analyze the main components of dissolved organic matter in the effluent. Figure 3 shows the fluorescence intensity contours of the effluent from Reactors 1 and 2. As shown in Figure 3a,b, no peaks were found in the EEM spectrum of Reactor 2 effluent, while two main peaks (Peaks A and B) were observed in Reactor 1 effluent. Peak A is characterized by an emission/absorbance of energy at an Ex/Em wavelength pair of 270/330–340, and Peak B at 230/330–340. According to the classification diagram of Chen et al. [30], Peak A is located in region IV and is associated with soluble microbial products (SMP)-like, predominantly protein-derived compounds. Peak B is located in region II and is associated with aromatic protein, mainly tryptophan. Therefore, the effluent from Reactor 1 mainly contained protein-like and SMP-like substances, corresponding to the slightly higher COD_Cr_. A similar phenomenon was also found by Chu and Wang [15] using polyurethane foam and biodegradable polymer as carriers in moving biofilm reactor for nitrate removal

To explore the effects of consumption rate and related organic carbon on denitrification, the mass balance of TOC in solid-phase denitrification biofilter using PCL as biofilm carrier and carbon source was studied and the results are presented in Table 2. As can be seen in Table 2, the organic carbon released from PCL by biological degradation (*C_b_*) accounted for 82.84% of the TOC released from PCL (*C_p_* + *C_b_*), and the organic carbon consumed by denitrification (*C_d_*) accounted for 93.58% of the TOC released from PCL (*C_p_* + *C_b_*), suggesting that the organic carbon released from PCL was mainly caused by biological degradation and most of the organic carbon was consumed by biological denitrification.

### 3.2. Effect of HRT on Denitrification Performance

**Figure 4:**
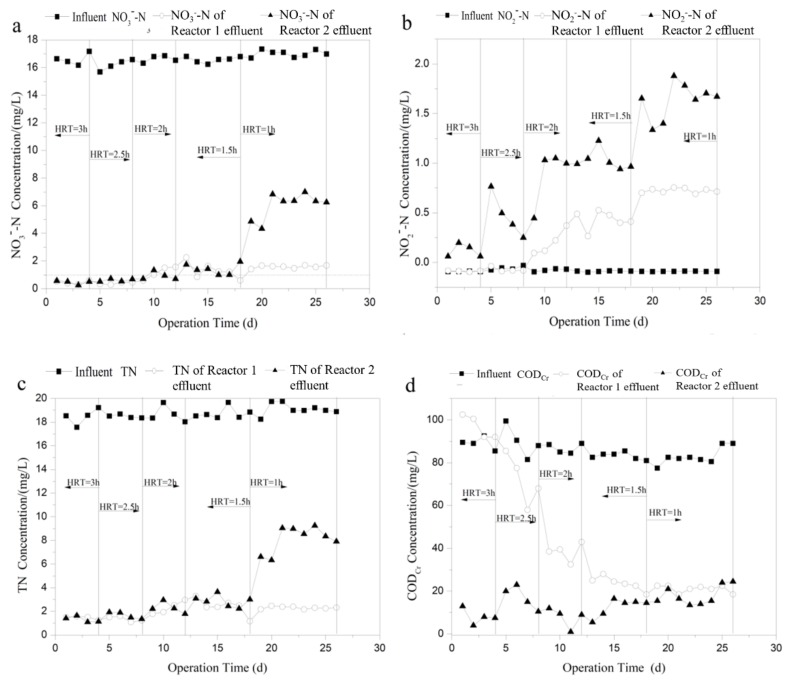
Influence of HRT on effluent: (**a**) nitrate; (**b**) nitrite; (**c**) TN; and (**d**) COD_Cr_ concentration of the two processes.

The two reactors were operated at different retention times varying from 1 to 3 h to determine the effect of different HRT value on nitrogen removal efficiency. The influent COD_Cr_ and NO3−-N concentration were controlled at 80 mg/L and 17.5 mg/L, respectively. Figure 4 shows the change in effluent nitrate, nitrite, TN and COD_Cr_ concentration over time. As shown in Figure 4a,c, high denitrification performance was achieved in the two reactors at the HRT of 3 h, and the nitrate removal efficiency was 96.6–98.4%. When HRT decreased from 3 to 1.5 h, NO3−-N and TN concentration in the effluent of the two reactors did not change significantly, and the average NO3−-N and TN concentration increased slightly from 0.5 and 1.3 mg/L to 1.3 and 2.6 mg/L. At Day 18, HRT changed from 1.5 to 1 h, while NO3−-N and TN in the effluent of Reactor 2 increased sharply. Then, NO3−-N and TN became almost constant, approximately 6.5 and 8.7 mg/L, respectively. However, NO3−-N and TN concentrations in the effluent of Reactor 1 remained at a low level. Thus, it can be concluded that the influence of HRT on Reactor 1 was limited, and high nitrogen removal efficiency was achieved even under low HRT value. 

The effect of HRT on nitrite accumulation and effluent COD_Cr_ concentration in the two reactors are illustrated in Figure 4b,d. As can be seen in Figure 4b, nitrite concentration in the effluent of Reactors 1 and 2 gradually increased as the HRT decreased, and the nitrite accumulation in Reactor 2 was more obvious than that of Reactor 1, suggesting that the second step of denitrification of reducing NO2−-N into N_2_ was severely inhibited. The shorter HRT value resulted in a decrease in nitrate removal and an increase in nitrite accumulation in Reactor 2 mainly because of the low contact time for microbial activity [31]. Under high flow rate condition, the soluble organic carbon was quickly washed out along with the effluent before it can be utilized by the denitrifiers. This was confirmed by the change of COD_Cr_ concentration in the effluent of Reactor 2. As can be seen in Figure 4d, COD_Cr_ concentration in the effluent of Reactor 2 increased obviously from 9.0 mg/L at 2 h to 24.5 mg/L at 1 h. However, COD_Cr_ concentration in the effluent of Reactor 1 decreased with the decrease of HRT. The result is in agreement with the previous studies showing that effluent dissolved organic carbon (DOC) increases by two times at the HRT of 2 h compared to that at the HRT of 7 h using ethanol as carbon source, while DOC decreases with increasing flow rate in denitrification system using solid substrate as carbon source [20,32]. 

**Figure 5:**
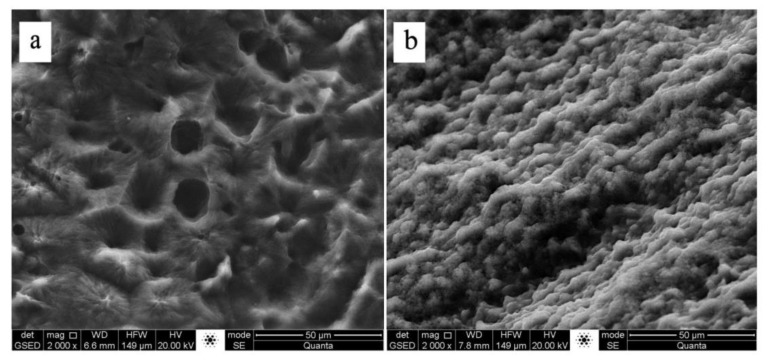
ESEM observation of: (**a**) surface of raw PCL; and (**b**) biofilm attached on PCL at HRT of 1 h.

Previous studies showed that biofilms can easily be washed away from PCL carrier, due to the greater shear force and large volume load caused by shorter Hydraulic retention time, significantly reducing the removal efficiency [15]. In addition, the biofilm on the surface of PCL carrier was responsible for roughly 92% of denitrification in the reactor using PCL as biofilm support and carbon source [11]. Therefore, the deterioration of denitrification performance was supposed to occur in Reactor 1 as the HRT decreased from 1.5 to 1 h. However, the nitrogen removal efficiency remained at a high level as the influent flow rate increased. This was probably due to the special physical structure of the PCL used in this study. Figure 5 presents the ESEM images of the PCL before and after biofilm attachment. As can be seen in Figure 5a, PCL carrier had many clear pores on its irregular and rough surface, and the pore structure could not only provide space for microorganism to grow and acclimatize, but also provide shelter for the biofilm to avoid being washed away by the high shear force under low HRT condition. This was further confirmed by the ESEM image of the surface of PCL at the HRT of 1 h. As shown in Figure 5b, under high shear force condition, biofilm was still firmly attached to the PCL carrier, able to take full advantages of carbon source for denitrification.

### 3.3. Effect of C/N Ratio on Denitrification Performance

**Figure 6:**
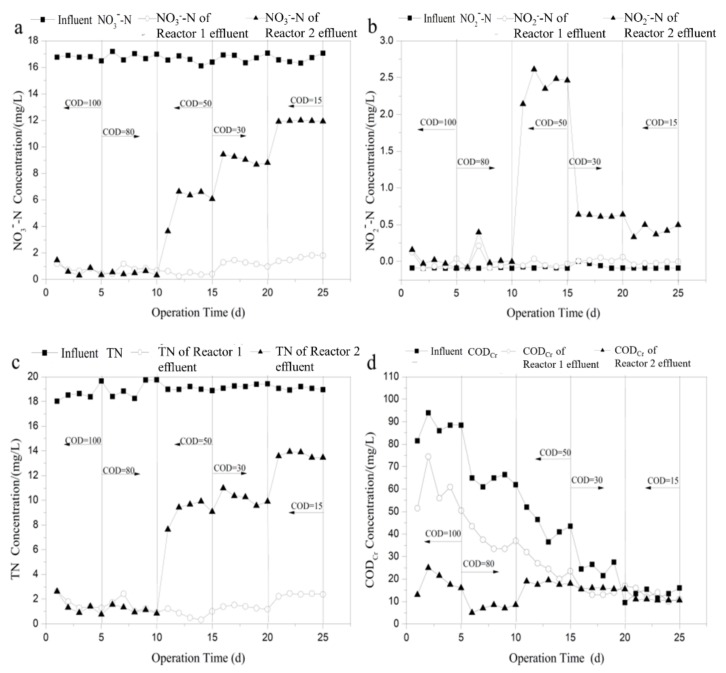
Influence of C/N ratio on effluent: (**a**) nitrate; (**b**) nitrite; (**c**) TN; and (**d**) COD_Cr_ concentration of the two processes.

C/N ratio was a very important factor for nitrogen removal [33]. To explore the influence of C/N ratio on the nitrogen removal performance, the two reactors were operated under different C/N ratio varying from 0.9 to 6, with 2.5 h of HRT at constant NO3−-N concentration of 16.7 mg/L. The effect of changing C/N ratio on effluent nitrate, nitrite, TN and COD_Cr_ concentration of two biofilters are shown in Figure 6. As shown in Figure 6a,c, both reactors exhibited high denitrification performance when C/N ratio was higher than 4.8, and the average concentration for NO3−-N and removal rate for TN of the effluent of Reactors 1 and 2 were 0.8 mg/L and 91.6%, and 0.6 mg/L and 93.1%, respectively. This result is consistent with previous studies showing that the best denitrification performance is obtained with a C/N ratio of 4–5, using sodium acetate as the sole source of carbon [34]. With the decrease of C/N ratio, however, Reactors 1 and 2 presented totally different nitrogen removal performance. When C/N ratio decreased from 4.8 to 0.9, the average concentrations for NO3−-N and TN of the effluent of Reactor 2 increased from 0.6 mg/L and 1.3 mg/L to 12.0 mg/L and 13.7 mg/L, respectively, while those in the effluent of Reactor 1 remained at a low level during the whole experimental period. The average concentration for NO3−-N and TN of the effluent of Reactor 1 were less than 1.6 mg/L and 2.4 mg/L even when the C/N ratio was 0.9. As discussed above, most of the organic carbon released from PCL was caused by biological degradation, and most of the organic carbon was consumed by biological denitrification. When the C/N ratio decreased, denitrifying microorganisms could obtain carbon through the enzymatic degradation of PCL, and the organic carbon released from PCL could also eliminate the dissolved oxygen in the influent to maintain the anoxic environment. However, ceramsite carrier in Reactor 2 could not provide organic carbon by itself, and the denitrification performance of Reactor 2 mainly relied on the organic carbon in the influent. When the C/N ratio decreased, organic carbon in the influent was rapidly oxidized to CO_2_ by dissolved oxygen in the influent, instead of providing electron donor for denitrification.

As can be seen in Figure 6b, when C/N ratio was higher than 4.8, nitrite concentration in the effluent of Reactors 1 and 2 were kept at a very low level, indicating that influent carbon source can meet the requirement of complete denitrification. However, with the decrease of C/N ratio, nitrite accumulation increased obviously at first and then decreased gradually in the effluent of Reactor 2, suggesting that nitrate reduction process was inhibited at first and then nitrite reduction process was inhibited. Nitrite concentration in the effluent of Reactor 1 was below 0.5 mg/L during the whole experimental period, probably due to the continuous and stable organic carbon source provided by the biodegradable polymer carrier. This was further confirmed by the COD_Cr_ change in the effluent of Reactors 1 and 2. As shown in Figure 6d, COD_Cr_ concentration in the effluent of Reactor 2 was higher than that of Reactor 1 during the whole experiment process.

**Figure 7:**
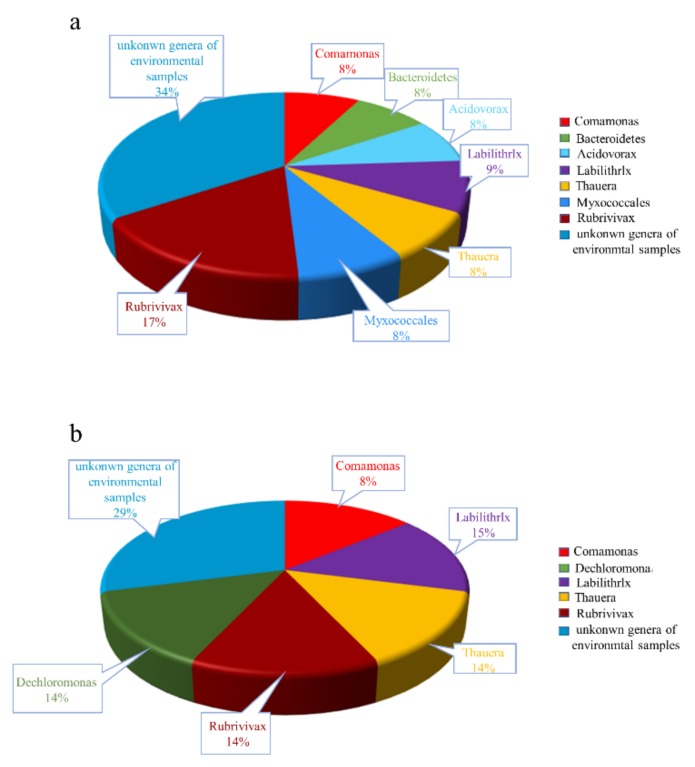
Distribution of microbial community a genus level: (**a**) Reactor 1; and (**b**) Reactor 2.

In addition, the difference in nitrogen removal efficiency between the two reactors under low C/N condition could partially be explained by the different microbial composition in the two reactors. Figure 7 shows the distribution of microbial community at genus level in the two reactors. As shown in Figure 7b, *Thauera* and *Dechloromonas* were observed in Reactor 2. This is consistent with previous studies showing that, in activated sludge, *Thaurea* and *Dechloromonas* are the dominant denitrifying bacteria adapted to an external carbon source such as acetate and methanol [35,36]. Since acetate in the influent was the sole carbon source for denitrification, the nitrogen removal efficiency of Reactor 2 declined gradually as the influent C/N decreased. In Reactor 1, however, the microbial composition was quite different from that of Reactor 2. As can be seen in Figure 7a, in addition to *Thaurea*, *Myxococcales, Rubrivivas, Acidovorax* and *Commanoas* were also observed in Reactor 1 using biodegradable polymers as biofilm carrier and solid carbon source. *Myxobacteria*, which feed on insoluble organic substrate, was found to be the most active denitrifiers in soddy podzolic soil and the maximum denitrifying activity was demonstrated by *Myxobacteria* [37]. Previous studies showed that *Rubrivivas gelatinosus* can reduce nitrite to N_2_, but cannot use nitrate as electron acceptor [38]. *Comamonas granuli*, which belongs to Comamonadaceae, was proved to be positive for nitrate reduction to nitrite [39], which is consistent with a previous study showing that the predominant PCL-degrading denitrifying bacteria are assigned to the family Comamondacea [12]. Therefore, high nitrogen removal efficiency obtained under low C/N ratio in Reactor 1 was probably due to the biological degradation of PCL by some of the above mentioned denitrifiers to acquire the necessary electron donor for denitrification. The comparison of the biomass contents of the biofilm attached to the two carriers showed that the biofilm’s VSS of PCL (0.20 VSS g/L) was close to that of ceramsite (0.22 VSS g/L), contrary to a previous study showing that the biomass on PCL was much lower that on polyurethane (PU) [15]. The possible explanations for the difference were that the PCL carrier used in this study can be degraded by microorganisms to form irregular shape and pore structure instead of solid shape and smooth surface, which gave it a large specific surface area (PCL carrier has a specific surface area of 11.4 m^2^/g, which is much larger than that of the clay ceramsite carrier of 5.6 m^2^/g). Thus, PCL carrier was favorable for the biofilm attachment, and the obvious decrease of biofilm thickness can mainly be explained with the lower substrate condition (C/N ratio = 0.9) [7].

### 3.4. Effect of Shock Loading of Influent Nitrate on Denitrification Performance

**Figure 8:**
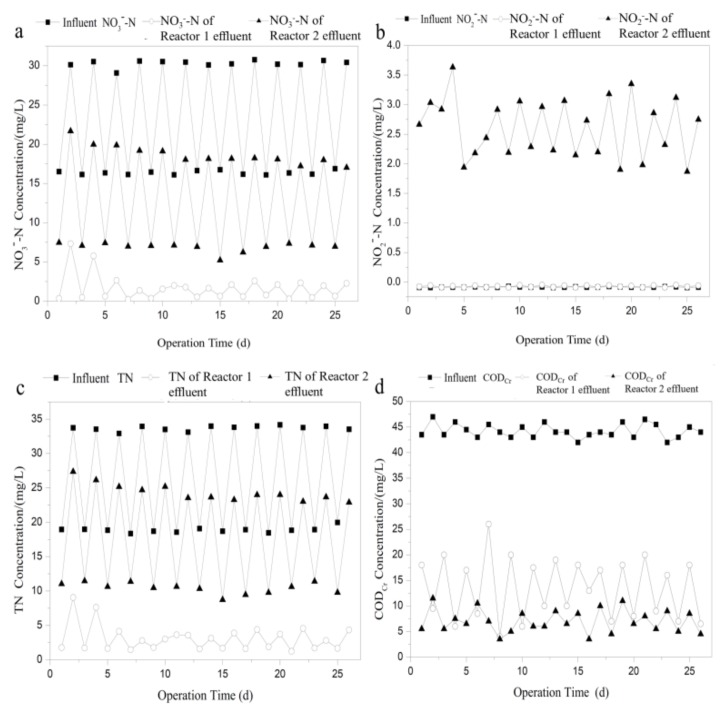
Influence of nitrate shock loading on effluent: (**a**) nitrate; (**b**) nitrite; (**c**) TN; and (**d**) COD_Cr_ concentration of the two processes.

To investigate the influence of continuous influent nitrate impact loading on the denitrification performance of the two reactors, NO3−-N concentration in the influent was changed between 16.1 and 30.8 mg/L, alternatively, with 2.5 h of HRT at constant influent COD_Cr_ concentration of 50 mg/L. Figure 8 depicts the influence of nitrate shock loading on effluent nitrate, nitrite, TN and COD_Cr_ concentration of the two reactors. As shown in Figure 8a,c, the concentration changes of NO3−-N and TN in the effluent and influent of Reactor 2 showed relatively similar trends, both fluctuating jaggedly. The range of the concentrations of NO3−-N and TN in the effluent of Reactor 1 fluctuated greatly within 6.9–18.7 mg/L and 10.4–24.3 mg/L, respectively. Concentrations of NO3−-N and TN in the effluent of Reactor 1, however, changed slightly at the beginning and became stable from Day 5, and the average effluent NO3−-N and TN concentrations were 1.4 and 2.8 mg/L, respectively. Therefore, it can be concluded that the biofilter using biodegradable polymer as biofilm carrier and carbon source can endure higher nitrate shocking than that of traditional ceramsite filter. Zhu et al. [40] also reported that a denitrification reactor using PBS as biofilm carrier and carbon source can resist a varying nitrate load, and the operation of reactor can remain stable even if the influent nitrate is sharply increased.

This deduction was further confirmed by the change of NO2−-N concentration in the effluent of the two reactors. As can be seen in Figure 8b, under the condition of continuous nitrate impact loading, NO_2_−-N in the effluent of Reactor 1 fluctuated greatly while NO2−-N in the effluent of Reactor 2 was not detected during the whole experimental period. It is interesting to note that the COD_Cr_ concentration in the effluent of Reactor 2 was kept at a low level during the whole process, while the COD_Cr_ concentration in the effluent of Reactor 1 had a negative relationship with the influent nitrate concentration (Figure 8d). Previous studies showed that the biological degradation of solid carbon source and denitrification are two independent processes [41]. Therefore, the high/low COD_Cr_ concentration at low/high influent nitrate (16.1 and 30.8 mg/L) could possibly be explained by the continuous degradation without/with full utilization of denitrifying bacteria.

## 4. Conclusions

Solid-phase denitrification biofilter and conventional denitrification biofilter exhibited the equivalent performance on acclimation time. However, solid-phase denitrification biofilter is more conductive to domestication of denitrifying microorganisms under the high level of influent nitrate load, greatly enhancing the nitrogen removal efficiency. Moreover, shorter HRT in solid-phase denitrification process further enhanced nitrogen removal efficiency since the biofilm was firmly attached to the clear pores on the irregular and rough surface of PCL carrier. Under the low C/N ratio, nitrogen removal efficiency of PCL carrier is higher than ceramsite carrier, owing to the former having *Commanoas*, which can degrade PCL carrier to produce electron for denitrification. Solid-phase denitrification process also had a stronger resistance of shock loading when compared to conventional denitrification. For treating wastewater with a low C/N ration, the Pack-bed Biofilm Reactor filled with biodegradable PCL carrier showed better performance.

## Figures and Tables

**Table 1:** Physico-chemical parameters of the two biofilm carriers in this study.

Carrier type	Product mark	Appearance shape	Density (g/mL)	Diameter (mm)	Height (mm)	Molecular Weight (Dalton)
Clay Ceramsite	PP-B 3.0	pellet	1.67	4–6	-	-
PCL	1400C	cylinder	1.08	3	4	140,000

**Table 2:** Mass balance table of TOC in solid-phase denitrification biofilter.

Average Water Quality in Influent and Effluent/(mg·L^−1^)		Calculation Results/(mg·d^−1^)	
Influent NO3−-N	48.79	Denitrification Consumption (Cd)	987.96
Effluent NO3−-N	0.74	DO Consumption (*C_o_*)	24.54
Inffluent DO	6.66	TOC Residual (*C_r_*)	43.28
Effluent DO	3.25	Physical Dissolution (*C_p_*)	182.0
Influent TOC	4.80	Biological Degradation (*C_b_*)	873.78
Effluet TOC	6.60		
